# Hoarseness as an Early Manifestation of Granulomatosis with Polyangiitis: A Case Highlighting Diagnostic Challenges

**DOI:** 10.7759/cureus.110622

**Published:** 2026-06-10

**Authors:** Adriana M Gomez, Carlos J Palencia, Carlos Flores Chique

**Affiliations:** 1 Internal Medicine, Spartanburg Medical Center, Spartanburg, USA; 2 Internal Medicine, Nephrology, Endocrinology, Rheumatology, University of Carabobo, Valencia, VEN; 3 Internal Medicine, Pontifical Catholic University of Chile, Santiago, CHL

**Keywords:** anca-associated vasculitis, granulomatosis with polyangiitis (gpa), hoarseness, laryngeal involvement, rapidly progressive glomerulonephritis (rpgn)

## Abstract

Granulomatosis with polyangiitis (GPA) is a systemic necrotizing small-vessel vasculitis that commonly involves the upper and lower respiratory tracts and kidneys and is potentially life-threatening. Early manifestations may be subtle and misleading, resulting in delayed diagnosis and treatment. Laryngeal involvement, particularly presenting as isolated dysphonia, remains an underrecognized and easily overlooked feature.

We report a 77-year-old man with a history of lymphoma and previous lung cancer in remission who presented with six weeks of progressive hoarseness, shortness of breath, profound weight loss, and constitutional symptoms. On presentation, he was found to have multifocal pulmonary opacities, acute kidney injury with active urinary sediment, and systemic inflammation. The constellation of symptoms and his complex medical history created a diagnostic challenge. A unifying diagnosis emerged only after serologic testing revealed positive cytoplasmic anti-neutrophil cytoplasmic antibodies (C-ANCA) and anti-elevated proteinase-3 (PR3) antibodies. Renal biopsy confirmed pauci-immune crescentic glomerulonephritis consistent with GPA. Prompt initiation of pulse-dose corticosteroids and rituximab resulted in rapid clinical improvement.

This case underscores dysphonia, which may represent an early and critical clue to airway involvement, preceding fulminant multisystem disease. Recognition of this presentation is essential, as diagnostic delay may lead to irreversible organ damage, whereas timely immunosuppressive therapy can be rapidly life-saving and organ-preserving.

## Introduction

Granulomatosis with polyangiitis (GPA) is a rare, potentially life-threatening necrotizing vasculitis of small- and medium-sized vessels that belongs to the spectrum of anti-neutrophil cytoplasmic antibody (ANCA)-associated vasculitides (AAV) [1]. The annual incidence is estimated at approximately 10-20 cases per million individuals, with peak onset occurring between the fifth and seventh decades of life [1,2]. GPA is characterized by granulomatous inflammation and vasculitis predominantly affecting the upper and lower respiratory tracts and kidneys, although virtually any organ system may be involved [2,3]. Disease pathogenesis is strongly associated with ANCA directed against proteinase-3 (PR3), which promotes neutrophil activation, endothelial injury, and subsequent vascular inflammation [1,3].

The classic clinical presentation consists of sinonasal disease, pulmonary involvement, and rapidly progressive pauci-immune glomerulonephritis; however, GPA exhibits substantial clinical heterogeneity and may initially present with isolated or atypical manifestations [2,4]. Upper airway involvement occurs in approximately 70-90% of patients during the disease course and frequently represents one of the earliest sites of disease activity [3,5]. Common manifestations include chronic rhinosinusitis, nasal crusting, epistaxis, otologic symptoms, and, less commonly, laryngotracheal disease [4,5].

Laryngeal involvement is an uncommon but clinically important manifestation of GPA, reported in approximately 10-20% of patients and most often involving the subglottic region [5-7]. Presenting symptoms may include hoarseness, dysphonia, cough, dyspnea, stridor, or airway compromise, often mimicking more common conditions, such as infection, malignancy, chronic obstructive pulmonary disease, or vocal cord dysfunction [5-8]. Consequently, diagnosis may be delayed, particularly in older individuals with significant smoking histories or pre-existing pulmonary disease. Recognition of these atypical presentations is critical because delayed diagnosis can result in irreversible organ damage and increased morbidity [2,4].

We present the case of a 77-year-old man with PR3-ANCA-positive GPA whose initial and predominant manifestation was progressive dysphonia, preceding the development of rapidly progressive glomerulonephritis, inflammatory arthritis, pulmonary abnormalities, and constitutional symptoms. This case highlights the diagnostic challenges posed by atypical laryngeal presentations of GPA and emphasizes the importance of maintaining a high index of suspicion for systemic vasculitis in patients with unexplained voice changes and evolving multisystem disease.

## Case presentation

A 77-year-old man with a significant past medical history of essential hypertension, hyperlipidemia, lymphoma, and stage IB lung cancer status post-resection and adjuvant therapy (in 2019) presented with a six-week history of progressive dysphonia characterized by hoarseness and loss of vocal strength. This was accompanied by profound constitutional symptoms, including an unintentional 30-pound weight loss, night sweats, generalized weakness, and anorexia.

Four weeks before presentation, the patient was hospitalized at an outside institution due to complaints of shortness of breath, and was found to be in acute decompensated heart failure and atrial fibrillation with rapid ventricular response. The report of the transthoracic echocardiography performed at that facility described a reduced ejection fraction (35%), global hypokinesis, and a left atrial appendage thrombus; images were not available. He was also treated empirically for pneumonia in the setting of bilateral pulmonary infiltrates. Management included broad-spectrum antibiotics, diuresis, anticoagulation with warfarin, and initiation of guideline-directed medical therapy for heart failure, including spironolactone, torsemide, carvedilol, and dapagliflozin. Iron deficiency anemia was also identified without overt bleeding. Notably, his dysphonia was already present during that admission but was not evaluated.

On presentation to our institution, the patient appeared cachectic with marked sarcopenia, including temporal and thenar muscle wasting, with a weak, breathy voice and bilateral rhonchi. He was otherwise hemodynamically stable and well oxygenated on room air. Initial laboratory evaluation, as reported in Table 1, demonstrated leukocytosis with neutrophilia, normocytic anemia, thrombocytosis, and a markedly supratherapeutic international normalized ratio (INR: 8.4). Renal function was impaired (elevated blood urea nitrogen (BUN) and creatinine), with partial improvement following intravenous fluid resuscitation. Additionally, elevated procalcitonin and positive *Mycoplasma* serologies were present.

**Table 1:** Abnormal admission laboratory results INR: international normalized ratio; BUN: blood urea nitrogen

Test	Reference	Results
Procalcitonin	0.02-0.25 ng/mL	0.9 ng/mL
White blood cell count	4.0-11.0 x 10^3^/µL	16.8 x 10^3^/µL
Hemoglobin	11.5-15.0 g/dL	9.7 g/dL
Platelets	135-400 x 10^3^/µL	561 x 10^3^/µL
INR	2.0-3.0	8.4
BUN	7-23 mg/dL	87 mg/dL
Creatinine	0.70-1.30 mg/dL	3.43 mg/dL

Further workup in the emergency department included a CT angiography of the chest that showed no pulmonary embolism but demonstrated the presence of panlobular emphysema and left-sided consolidation, as shown in Figure 1. The patient was admitted with presumed left lobar pneumonia and started on empiric antibiotics with ceftriaxone and doxycycline, which were de-escalated to doxycycline monotherapy for seven days.

**Figure 1:**
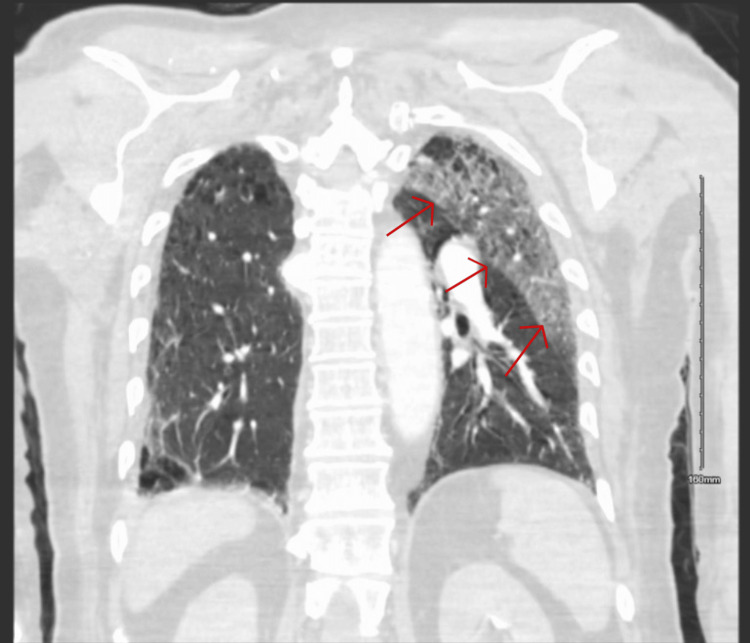
Computed tomography of chest (coronal view). Red arrows show consolidative changes in the left upper lobe. Diffuse emphysematous changes are present in both lungs.

CT imaging of the neck showed no structural abnormalities to account for his dysphonia, as shown in Figure 2. Speech-language pathologist evaluation revealed severe vocal weakness with limited phonation and reduced endurance; unfortunately, the patient declined fiberoptic endoscopic evaluation, and direct visualization of vocal cords was not otherwise possible. Workup for neuromuscular disease was otherwise unrevealing, with normal MRI of the brain (Figure 3) and cervical spine (Figure 4), preserved respiratory mechanics, and normal muscle markers. Outpatient otolaryngology follow-up was planned.

**Figure 2:**
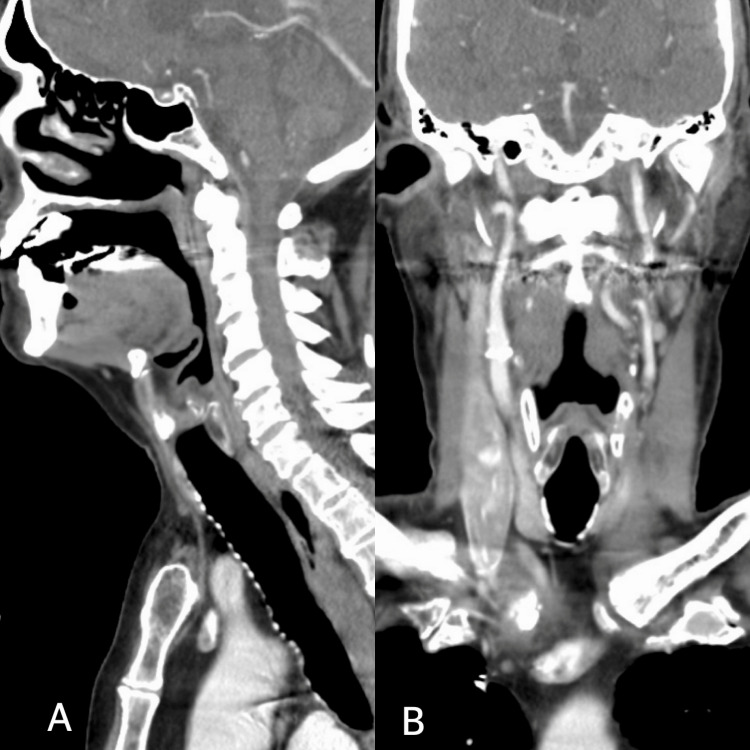
Computed tomography of the neck. Sagittal (A) and coronal (B) views of the neck showing no acute abnormalities.

**Figure 3:**
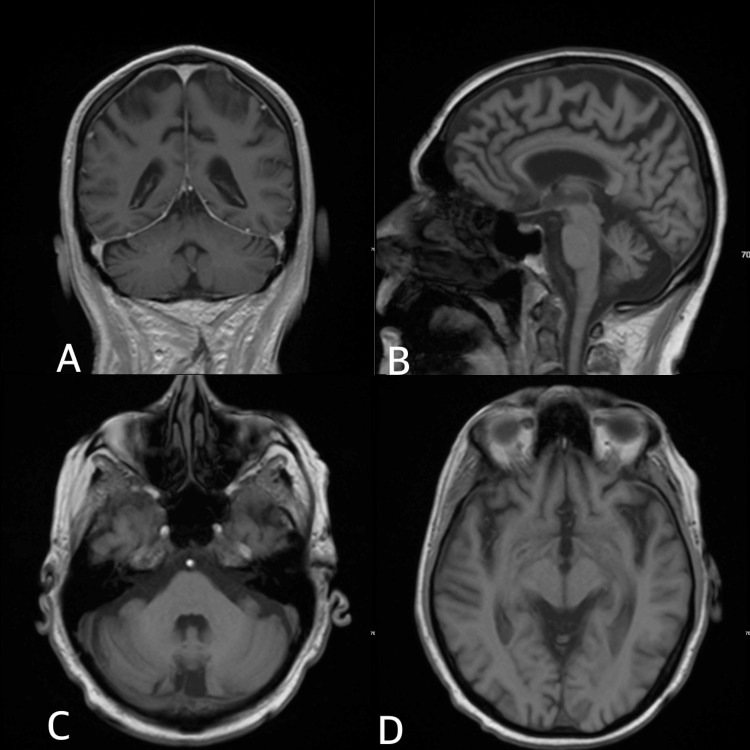
Magnetic resonance imaging (MRI) of the brain. (A) Coronal T1-weighted post-contrast image. (B) Sagittal T1-weighted image. (C) Axial T1-weighted image through the posterior fossa. Unremarkable cerebellar hemispheres and brainstem without focal abnormality. (D) Axial T1-weighted image at the level of the basal cisterns. A, B, C, and D images demonstrate no abnormal intracranial or extra-axial lesion, or preserved midline structures. Age-related mild generalized cerebral volume loss is present.

**Figure 4:**
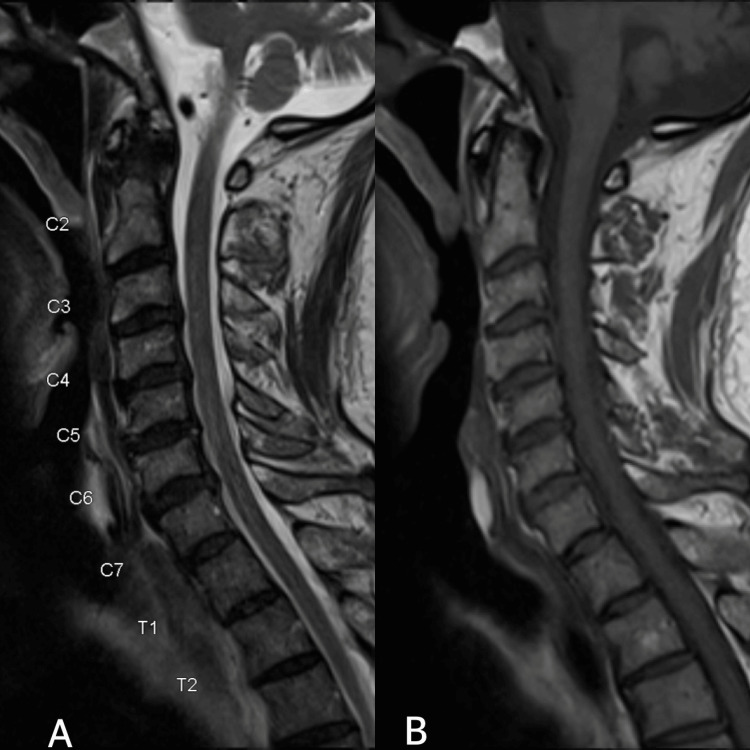
Magnetic resonance imaging (MRI) of the cervical spine. Sagittal T2-weighted (A) and sagittal post-contrast T1-weighted (B) MRI images of the cervical spine showing multilevel chronic degenerative changes without significant spinal stenosis, cord compression, or other acute lesions. MRI: magnetic resonance imaging

Given the association with his rapid weight loss and oncologic history, malignancy recurrence was considered; however, CT imaging of the chest, abdomen, and pelvis showed no evidence of mass or lymphadenopathy, as shown in Figure 1 and Figure 5.

**Figure 5:**
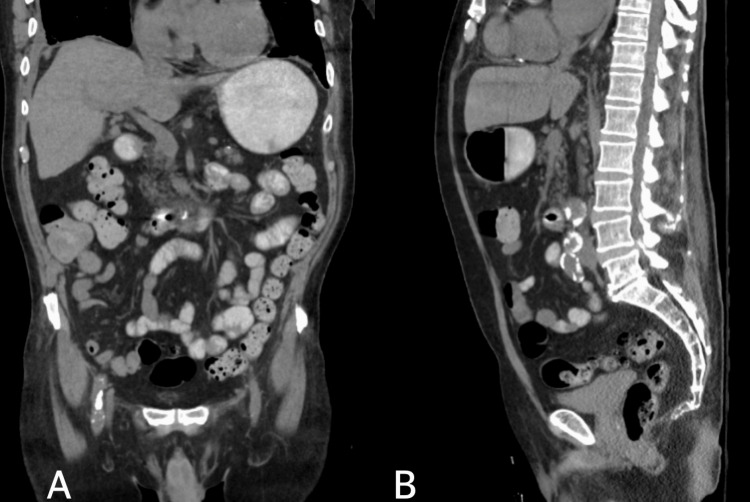
Computed tomography of the abdomen and pelvis. Coronal (A) and sagittal (B) images showing no gross evidence of metastatic disease.

This patient's renal function initially improved after fluid administration, suggesting a pre-renal etiology, but worsened subsequently (as shown in Table 2) despite adequate volume status and hemodynamics, opening the differentials, the recent exposure to multiple nephrotoxic medications (iodinated contrast, diuretics, and sodium-glucose cotransporter 2 (SGLT2) inhibitor therapy) was suspected to have contributed. Warfarin-related nephropathy was also considered, given the presentation with significantly elevated INR. Nephrology was consulted, and further workup was pursued: urinalysis revealed proteinuria and microscopic hematuria (>180 red blood cells (RBCs)/hpf), raising concern for intrinsic renal pathology. Renal ultrasound showed no obstruction. 

**Table 2:** Kidney function progression BUN: blood urea nitrogen

Test	Reference	Day 1	Day 2	Day 5	Day 8	Day 10
BUN	7-23 mg/dL	87 mg/dL	86 mg/dL	82 mg/dL	105 mg/dL	153 mg/dL
Creatinine	0.70-1.30 mg/dL	3.43 mg/dL	3.27 mg/dL	4.57 mg/dL	4.83 mg/dL	5.03 mg/dL

On hospital day 2, the patient developed acute migratory polyarthritis involving the wrists and shoulder without any previous trauma; imaging was unremarkable.

Given the constellation of progressive renal dysfunction, inflammatory arthritis, pulmonary findings, and constitutional symptoms, ANCA-associated vasculitis was suspected. High-dose intravenous methylprednisolone (500 mg daily for three days) was initiated empirically on hospital day 7, resulting in rapid resolution of joint symptoms and further increase in BUN/creatinine, which peaked at day 10 (Table 2). At this time, the patient was transitioned to oral prednisone (1 mg/kg/day), and the results of the serologic workup revealed positive anti-cytoplasmic ANCA (C-ANCA), titers 1:160), and anti-PR3 antibodies, confirming the diagnosis of GPA, followed by the initiation of induction therapy with rituximab (1 g intravenous (IV)). 

The patient’s clinical status improved significantly with immunosuppressive therapy, including recovery of renal function (as shown in Table 3), improved energy levels, and restored functional mobility.

**Table 3:** Progression of kidney function after initiation of induction therapy BUN: blood urea nitrogen

Test	Reference	Day 12	Day 13	Day 14-discharge	Day 21	One month post-discharge
BUN	7-23 mg/dL	137 mg/dL	120 mg/dL	107 mg/dL	55 mg/dL	38 mg/dL
Creatinine	0.70-1.30 mg/dL	4.05 mg/dL	3.08 mg/dL	2.06 mg/dL	1.86 mg/dL	1.52 mg/dL

After normalization of INR, a renal biopsy was performed on hospital day 14, which demonstrated necrotizing and crescentic pauci-immune glomerulonephritis with diffuse acute tubular injury with foci of necrosis and moderate tubular atrophy with interstitial fibrosis (30-40%). Two glomeruli were globally sclerosed, with six revealing cellular crescents and three with regions of fibrinoid necrosis. The findings were consistent with GPA with renal, pulmonary, and suspected laryngeal involvement.

After discharge, the patient received a second dose of rituximab 14 days after the initial infusion. Avacopan was initiated, and corticosteroids were tapered off as part of the induction regimen and transition to maintenance therapy. 

On subsequent outpatient follow-up visits, the patient reported a significant reduction in hoarseness, with noticeable improvement in voice strength and overall vocal quality. 

## Discussion

Laryngeal involvement in GPA is a recognized but often underappreciated manifestation of the disease. Although upper airway involvement is common, affecting up to 90% of patients during the disease course, clinically significant laryngeal disease occurs less frequently and is most commonly associated with subglottic stenosis [3,5-7]. The pathologic process is thought to result from granulomatous inflammation and vasculitic injury involving the laryngeal and tracheal mucosa, leading to edema, fibrosis, and eventual airway narrowing [5,6]. Symptoms are often nonspecific and include hoarseness, dysphonia, cough, dyspnea, wheezing, or stridor, which can mimic infectious, malignant, or obstructive pulmonary conditions and contribute to diagnostic delays [4-7].

Several studies have characterized the spectrum of laryngeal manifestations in GPA. Gelbard et al. demonstrated that laryngeal disease can affect multiple subsites, including the true vocal folds, supraglottis, glottis, and subglottis, with dysphonia representing one of the most common presenting complaints [5]. Similarly, Ahn et al. reported that subglottic stenosis may occur either at initial presentation or during disease progression and often requires multidisciplinary management involving systemic immunosuppression and, in some cases, airway intervention [6]. Pakalniskis et al. further emphasized the diverse head and neck manifestations of GPA and highlighted the importance of imaging and clinical correlation when evaluating atypical upper airway symptoms [7].

Our case differs from many previously reported presentations because progressive dysphonia was the dominant and persistent symptom preceding recognition of systemic disease. While hoarseness has been described in association with GPA, airway manifestations are often identified after diagnosis or occur concurrently with more recognizable features such as chronic sinonasal disease, pulmonary nodules, or established renal involvement [4-7]. In contrast, our patient's voice changes prompted repeated evaluations and were initially attributed to multifocal pneumonia and other cardiopulmonary conditions. This diagnostic trajectory illustrates the substantial overlap between GPA and more common infectious or pulmonary disorders, particularly in elderly patients with significant oncologic and smoking histories.

The subsequent development of pauci-immune crescentic glomerulonephritis, inflammatory arthritis, constitutional symptoms, positive cytoplasmic ANCA serology, and elevated anti-PR3 antibody levels ultimately established the diagnosis of systemic GPA. Renal involvement occurs in approximately 70-80% of patients and remains one of the principal determinants of long-term morbidity and mortality [2,3]. The presence of rapidly progressive renal dysfunction in our patient underscored the aggressive nature of the disease and necessitated the prompt initiation of immunosuppressive therapy. Importantly, treatment with high-dose corticosteroids followed by rituximab resulted in stabilization of systemic disease, consistent with current evidence supporting rituximab as an effective induction agent for severe ANCA-associated vasculitis [8,9].

An additional feature of this case was the absence of direct laryngoscopic confirmation of airway involvement because invasive evaluation was deferred according to patient preference. Although tissue diagnosis and endoscopic assessment remain valuable diagnostic tools, real-world clinical decision-making often requires integration of serologic, radiographic, histopathologic, and clinical findings when complete airway evaluation is not feasible. This scenario reflects a common challenge in the diagnosis of GPA and highlights the importance of maintaining diagnostic flexibility when evaluating complex multisystem presentations.

This case reinforces the broad clinical spectrum of GPA and highlights progressive dysphonia as a potentially misleading early manifestation of systemic vasculitis. In patients presenting with persistent unexplained hoarseness, particularly when accompanied by constitutional symptoms, inflammatory arthritis, pulmonary abnormalities, or renal dysfunction, clinicians should maintain a broad differential diagnosis that includes ANCA-associated vasculitis. Early recognition of atypical airway manifestations may expedite diagnostic evaluation, facilitate timely initiation of immunosuppressive therapy, and reduce the risk of irreversible end-organ damage.

## Conclusions

Laryngeal involvement in GPA is an uncommon but recognized manifestation that may present with hoarseness, dysphonia, vocal cord dysfunction, or subglottic stenosis, sometimes preceding the development of more classic systemic features. This case highlights GPA presenting with the uncommon initial manifestation of progressive dysphonia, later found to be associated with multisystem involvement, including pauci-immune crescentic glomerulonephritis, pulmonary disease, inflammatory arthritis, and constitutional symptoms. The diagnosis was initially obscured by several competing clinical considerations, including pneumonia, heart failure, medication-related kidney injury, and possible malignancy recurrence, contributing to diagnostic complexity and delayed recognition. The constellation of symptoms ultimately raised suspicion for ANCA-associated vasculitis. The diagnosis was confirmed by positive PR3-ANCA serologies and renal biopsy and further supported by the significant clinical improvement following treatment with corticosteroids and rituximab.

This case underscores the importance of maintaining a broad differential diagnosis in patients presenting with unexplained dysphonia accompanied by systemic inflammatory manifestations and progressive kidney injury. Early recognition of atypical presentations of GPA is critical, as delayed diagnosis may result in irreversible end-organ damage, particularly renal impairment, and highlights the importance of prompt initiation of immunosuppressive therapy to improve clinical outcomes and preserve organ function.
